# Molecular detection and phylogenetic analysis of porcine circovirus type 3 in Tibetan pigs on the Qinghai-Tibet Plateau of China

**DOI:** 10.1186/s12985-022-01792-4

**Published:** 2022-04-07

**Authors:** Yangyang Pan, Shantong Qiu, Rui Chen, Tiantian Zhang, Linfeng Liang, Meng Wang, Abdul Rasheed Baloch, Libin Wang, Qian Zhang, Sijiu Yu

**Affiliations:** 1 College of Veterinary Medicine, Gansu Agricultural University, Lanzhou, 730070 Gansu China; 2 Dr. Panjwani Center for Molecular Medicine and Drug Research, International Center for Chemical and Biological Sciences, University of Karachi, Karachi, 75270 Pakistan

**Keywords:** PCV3, Tibetan pigs, Phylogenetic analysis, Genotypes, Pathogenicity

## Abstract

**Background:**

Porcine circovirus type 3 (PCV3) has been confirmed to infect pigs, posing a health risk and making pigs more susceptible to other pathogens. After the first report of PCV3 infection in the United States, its prevalence was determined in pigs suffering from clinical digestive or respiratory diseases in several other regions, including the Sichuan and Gansu provinces of China. In this study, we describe the frequency of PCV3 detection in Tibetan pigs inhabiting three different provinces surrounding the Qinghai-Tibet Plateau of China.

**Methods:**

A total of 316 samples from diarrheic animals and 182 samples from healthy animals were collected in a randomized manner. Conventional PCR was applied for PCV3 DNA detection. The conserved regions of the PCV3 gene were analyzed with MEGA 7.1 software to design specific primers to sequence entire Cap genes in PCV3 strains, and the sequences were then used to confirm the subtypes of PCV3 in the positive samples. Prediction of the amino acid sequences by nucleotide sequence translation was also performed to compare the point mutations in the entire Cap protein. Twenty PCV3 whole-genomic sequences were used for genome phylogenetic analyses of PCV3 and sequence alignments with 22 other reference strains.

**Results:**

We found that the prevalence of the virus was significantly higher in samples from pigs with diarrhoea than that in samples from healthy pigs. Phylogenetic analysis of Cap proteins demonstrated that the 20 PCV3 strains formed three clades, including PCV3a (8/20, 40.00%), PCV3b (5/20, 25%) and PCV3c (7/20, 35.00%). The complete genome sequence revealed that these strains formed one branch in the phylogenetic tree. Sequence analysis showed that the Cap proteins of the 20 different viral strains shared between 95.84 and 99.18% nucleotide identity. Cap protein sequence analyses showed that the positivity rate of PCV3a was highest in the samples from pigs with diarrhoea. In comparison, PCV3c was the most elevated subtype in the healthy samples. There was no mutation at a specific site in the amino acid sequences of the entire Cap protein from different PCV3 subtype strains from heathy samples. There was a mutation at site 113 in PCV3a, site 129 in PCV3b, and site 116 in PCV3c.

**Conclusion:**

Our present data provide evidence that PCV3 is prevalent in Tibetan pigs at high altitudes in China, and the higher prevalence rates of the PCV3a and PCV3b subtypes in samples from pigs with diarrhoea further indicate that the genotypes should not be neglected during surveys of the pathogenicity of PCV3. Phylogenetic and genetic diversity analyses suggested that the continuous evolution, adaptation and mechanisms of pathogenicity of PCV3 in Tibetan pigs living in this special environment should be further studied.

## Background

Porcine circoviruses (PCVs) are small non-enveloped DNA viruses with a circular single-strand DNA and belong to the genus Circovirus in the family Circoviridae [1, 2]. Four species of PCVs have been identified, i.e., the non-pathogenic PCV type 1 (PCV1), the pathogenic PCV type 2 (PCV2), PCV type 3 (PCV3) and PCV type 4 (PCV4) [3, 4]. Among the four types of PCVs, PCV1 was first confirmed as a contaminant of PK-15 cell cultures and it is not associated with clinical disease in pigs [5]. PCV2 infection causes various clinical diseases, resulting in substantial economic losses in the swine industry [6]. PCV3 was first identified in 2015 by metagenomics in pigs with porcine dermatitis nephropathy syndrome (PDNS), reproductive failure with cardiac arrest and multisystemic inflammation [7]. PCV4 was discovered as a new circovirus in April 2019 in pigs with severe clinical signs and was found to be distantly related to PCV3 [4, 8, 9].

PCV3, due to its high prevalence compared with other types of PCVs, has generated ample interest worldwide [10]. The PCV3 genome is 2000 bp in length, encodes a non-ATG-initiated replicase (Rep) and plays important roles in viral replication. The capsid (Cap) protein is a major antigen that performs a crucial function in inducing a specific immune response in its host, and is used as a phylogenetic and epidemiological marker due to its genetic variations [7]. Currently, PCV3 can be divided into at least three clades (PCV-3a, PCV-3b and PCV-3c) based on evolutionary analysis of cap genes [11]. After the first report of PCV3 infection in the United States [7], its prevalence was determined in pigs suffering from clinical digestive or respiratory diseases in several other countries, including Germany [12], Korea [13], Sweden [14], China [15], Russia [16] and Thailand [17]. However, to date, no epidemiological study of PCV3 has been conducted with healthy pigs as a control group on the same farm. Detection of PCV3 in Shandong Province of China showed that PCV3 was prevalent in pigs; however, intriguingly, the infected animals did not show any clinical signs [18]. These inconsistent results revealed that it is important to carry out further research to investigate the pathogenicity and prevalence characteristics of PCV3 in various parts of the world.

Due to the cold and harsh environment in the Qinghai-Tibet Plateau of China (altitude > 3000 m, average annual temperature < 0 °C), few infections with microorganisms have been reported in livestock to date [19, 20]. In 2018, we confirmed, for the first time, the presence of porcine deltacoronavirus (PDCoV) in Tibetan pigs suffering from diarrhoea [21], indicating the existence of viral infections in animals on the Qinghai-Tibet Plateau. To date, the PCV3 has been detected throughout commercial pig farms from 21 provinces/districts [22–24], including Sichuan and Gansu provinces of China [10], which are located near to the Qinghai-Tibet Plateau, suggesting that other microbial infections should not be ignored in these areas. We thus aimed to investigate whether PCV3 is prevalent in Tibetan pigs; and PCV3 genetic characterization was performed using intestinal tissues of healthy Tibetan pigs or those with digestive diseases of the Tibetan pigs from 56 farms from three provinces on the Qinghai-Tibet Plateau of China. The samples were collected from June 2018 to December 2019.

## Materials and methods

### Sample collection

We collected samples from the Tibetan pig farms located in the Sichuan, Gansu and Qinghai provinces, representing different areas on the Qinghai-Tibet Plateau. A total of 498 intestinal tissue samples from 56 farms were collected between June 2018 and December 2019. The collected samples included 316 diarrheic and 182 healthy animals. The PCV3 positivity rate was evaluated in Tibetan pigs with clinical signs of acute diarrhoea from 29 farms. Healthy control samples were collected in a randomized manner from 27 farms, based on 10% of the number of Tibetan in stock, and used to evaluate the latent PCV3 infection rate in healthy animals. All the sampled Tibetan pigs were at the weaner and grower stage, and were between 4 and 12 weeks old; no further categorization was performed on the basis of age. Intestinal tissues were stored in liquid nitrogen for DNA extraction. This study was granted approval from the veterinarians of the Animal Care and Use Committee at Gansu Agricultural University, China.

### DNA extraction and PCV3 detection

**Fig. 1:**
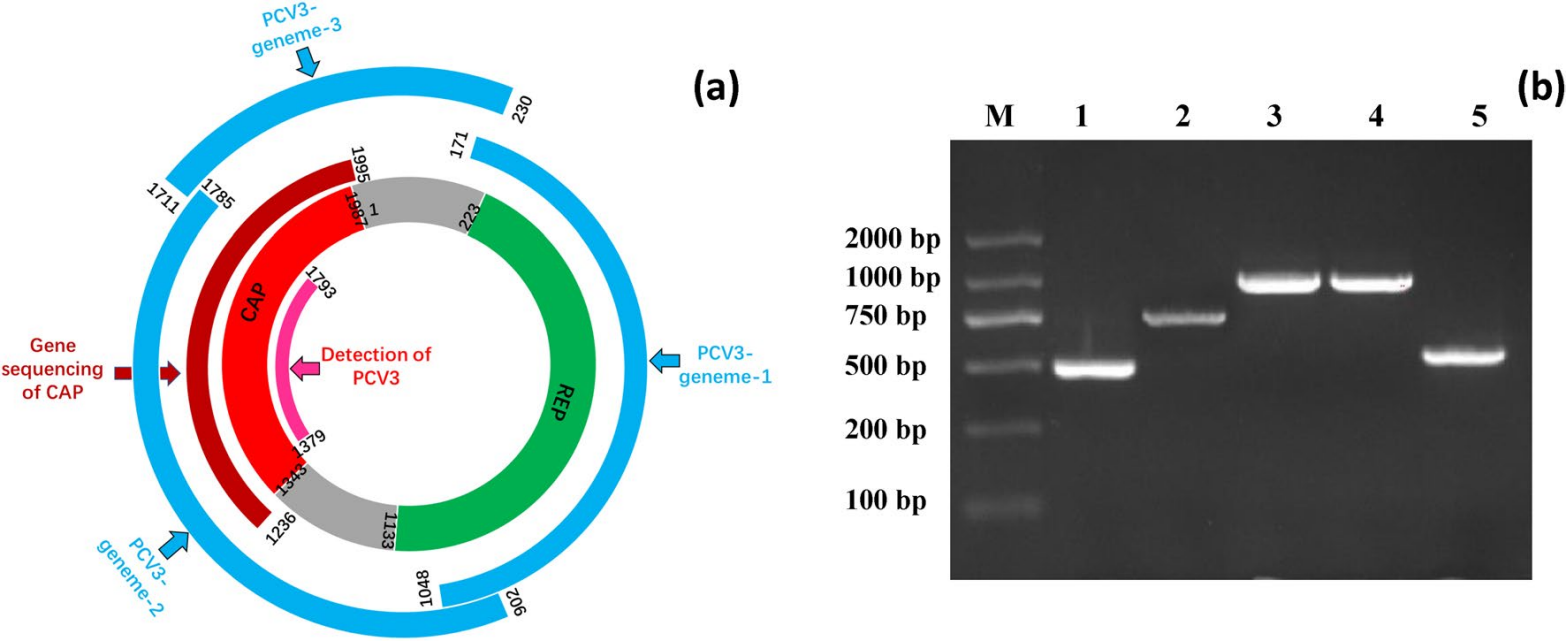
Design and PCR results of primers used to detect PCV3 and amplify Cap genes and full-length PCV3 genomes. **a** Design and PCR results of primers used to detect PCV3 and amplify Cap genes and full-length PCV3 genomes. **b** PCR results of primers used to detect PCV3 and amplify Cap genes and full-length PCV3 genomes, 1, 2, 3, 4, 5, PCR results for PCV3-D, PCV3-CAP, PCV3-genome-1, PCV3-genome-2, PCV3-genome-1, respectively

The intestinal tissues from Tibetan pigs were homogenized to powder with liquid nitrogen, and diluted threefold with phosphate‐buffered saline (PBS) in volume. All samples were centrifuged at 1800 × *g* for 10 min at 4 °C, and the supernatants were collected and transferred into a 1.5 mL tube. DNA extraction from supernatants was performed using a commercial DNA extraction kit (Omega, Norcross, GA, USA) according to the manufacturer’s instructions. The extracted sample DNA was stored at − 20 °C until being used for PCV3 detection. A pair of specific primers for PCV3 detection was designed based on the conserved sequence in the cap genes (Table 1, Fig. 1), and the expected size of the amplification product was 415 bp. The amplification reactions consisted of 1.0 μL of sample DNA, 1.0 μL of each primer at a concentration of 2.5 μM, 12.5 μL of 2 × PCR mix, and 9.5 μL of nuclease-free water added up to a final volume of 25 μL. The thermocycling program used was as follows: predenaturation at 95 °C for 5 min, followed by 35 cycles of 95 °C for 30 s, 58 °C for 25 s and 72 °C for 30 s, with a final extension for 10 min at 72 °C (Bio-Rad, USA). The PCV3 positivity rates in different provinces were calculated and marked on a map of China based on the electrophoresis results of the PCR products.

**Table 1:** Primers used for detection and full-length genome amplification of Porcine circovirus type 3 (PCV3) in Tibetan pigs

Primers	Sequences	Binding position^a^	Length^b^	Function^c^
PCV3-D-1379-F	5′-CCATTCGTTTAGGCGGGTAATG-3′	1379–1793	415	PCV3 detection
PCV3-D-1793-R	5′-CATTACCCGCCTAAACGAATGG-3′			
PCV3-CAP-1236-F	5′-ACATGCGAGGGCGTTTACCTGT-3′	1236–1995	760	Genome sequencing of capsid
PCV3-CAP-1995-R	5′-GCACCAARATGAGACACAGAGCT-3′			
PCV3-genome-1-F	5′-TGGTGCTACGAGTGTCCTGAAGAT-3′	171–1048	878	Amplify full-length PCV3 genomes
PCV3-genome-1-R	5′-CCTTRGTGAACCTCCTAAACAAGG-3′			
PCV3-genome-2-F	5′-ACCCTCTGAGGGTTCCTGTTAAG-3′	902–1785	884	
PCV3-genome-2-R	5′-GCCTAAACGAATGGGAAACTGC-3′			
PCV3-genome-3-F	5′-CAGGGCTGAGTGTAACTTTCATC-3′	1711–230	520	
PCV3-genome-3-R	5′-CACCGGACCTAGAATAGGATGA-3′

### Sequencing and analysis of Cap genes of PCV3 strains

The PCV3 strains PCV3/CN/Xinjiang-11/2018 and PCV3-China/GD-ZQ-1/2017 (MK284236 and MG897485) were chosen from GenBank, and the conserved regions of the PCV3 genes were analyzed with MEGA 7.1 software to design specific primers to sequence entire Cap genes in PCV3 strains, which were then used to confirm the subtypes of PCV3 in the positive samples. The references for the amplification reactions and PCR parameters are given in the methods in section of DNA extraction and PCV3 detection. The PCR products were purified and cloned using the pMD19-T vector (TaKaRa, Dalian, China) for sequencing (Sangon Biotech, Shanghai, China). Twenty different nucleotide sequences of Cap proteins were obtained from the PCR products and analyzed using the MegAlign program. Twenty-one different subtypes representative of PCV3 strains that occurred in different countries in recent years were chosen, and phylogenetic analysis of the entire Cap nucleotide sequences was performed using the neighbour‐joining method with 1000 bootstrap replicates in MEGA 7.2 software. To compare the number of mutations in the amino acid sequences of the entire Cap protein among the three PCV3 subtypes, different PCV3 strains and one reference strain with highest homology were chosen. Prediction of the amino acid sequence from nucleotide sequences translation was performed to compare the point mutations using MEGA and edited in Microsoft Excel, version 2016.

### Nucleotide sequence and whole-genome analyses of PCV3

Based on the phylogenetic analysis of the Cap nucleotide sequences, the DNAs from twenty positive samples with different Cap nucleotide sequences were subjected to PCR with three pairs of overlapping primers (Table 1, Fig. 1) to obtain the complete genome sequence of PCV3. Primers were designed based on conserved regions of the PCV3 strains PCV3/CN/Xinjiang-11/2018 and PCV3-China/GD-ZQ-1/2017 (MK284236 and MG897485). All the PCR assays were performed according to the methods described in section of DNA extraction and PCV3 detection. Three sequence fragments from one positive DNA sample were assembled and annotated using the EditSeq program of the DNASTAR software and aligned to obtain the final sequences of the PCV3 genomes. Twenty complete PCV3 genomic sequences in this study were used in phylogenetic analyses and sequence alignments with 22 other reference strains obtained in GenBank. Phylogenetic analysis of PCV3 whole-genome was performed using the neighbour‐joining method with 1000 bootstrap replicates in MEGA 7.2 software.

### Statistical analysis

All statistical tests were performed with IBM SPSS Statistics (v22.0), Student’s t test (dependent, two-tailed) was used to test for significant differences in the positivity rate among the groups. The significance level in all the analyses was 5%, with a confidence interval of 95%. Differences in values were considered significant at *P* < 0.05. The sequence for each of the PCR products in this study was performed using at least three replicates.

## Results

### Prevalence of PCV3 in Tibetan pigs

**Fig. 2:**
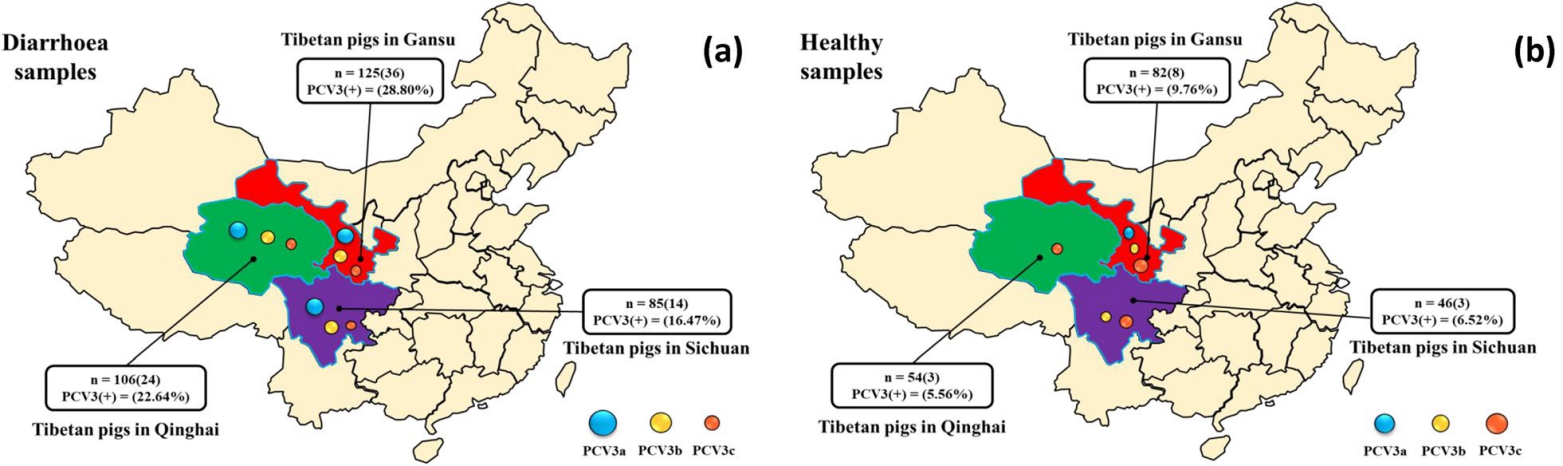
Geographic distributions of the identified porcine circovirus type 3 (PCV3) strains in Tibetan pigs from three provinces on the Qinghai-Tibet Plateau, China. **a** PCV3 in samples from diarrheic Tibetan pigs. **b** PCV3 in samples from healthy Tibetan pigs. “n” indicates the total number of samples in each province, and PCV(+) % indicates the PCV3 prevalence rate, with colour-coded circles used to distinguish subtypes of PCV3

In the present study, a total of 498 intestinal tissue samples from 56 Tibetan pig farms were collected from three different provinces on the Qinghai-Tibet Plateau of China. The collected samples were then subjected to PCV3 detection by using conventional PCR. The results showed that positivity rate for molecular detection of PCV3 in Tibetan pigs was 17.68% (88/498) at the sample level and 25.00% (14/56) at the farm level. Detailed information on 88 samples that were positive for PCV3 is shown in Fig. 2 and Table 2. The PCV3 positivity rate at the sample level was 21.25% (44/207) in Gansu, 16.88% (27/160) in Qinghai and 12.98% (17/131) in Sichuan. The PCV3 positivity rate at the farm level were 25.00% (6/24) in Gansu, 18.75% (3/16) in Qinghai and 31.25% (5/16) in Sichuan. The prevalence of PCV3 in samples from pigs with diarrhoea was significantly higher than that in samples from healthy pigs (sample levels: 23.42% vs. 7.69%; farmer levels: 27.59% vs. 2.22%). The PCV3 positivity rate in Gansu was found to be significantly higher than that in Sichuan and Qinghai provinces (Table 2).

**Table 2:** Results of porcine circovirus type 3 (PCV3) detection in tibetan pigs by polymerase chain reactions (PCRs) for three provinces surrounding Qinghai Tibetan plateau, China

Location (Province)	Diarrhoea Pos/Tot (%)								Healthy Pos/Tot (%)								Total Pos/Tot (%)	
	3a		3b		3c		Total Pos/Tot		3a		3b		3c		Total Pos/Tot			
	Farm level 95% CI	Sample level 95% CI	Farm level 95% CI	Sample level 95% CI	Farm level 95% CI	Sample level 95% CI	Farm level 95% CI	Sample level 95% CI	Farm level 95% CI	Sample level 95% CI	Farm level 95% CI	Sample level 95% CI	Farm level 95% CI	Sample level 95% CI	Farm level 95% CI	Sample level 95% CI	Farm level 95% CI	Sample level
Gansu	3/12 (25.00%) (0.023–0.049)	23/125 (18.40%) (0.015–0.132)	2/12 (16.67%) (0.020–0.036)	9/125 (7.20%) (0.028–0.052)	3/12 (25.00%) (0.006–0.425)	4/125 (3.20%) (0.120–0.450)	3/12 (25.00%) (0.086–0.333)	36/125 (28.80%) (0.204–0.470)	2/12 (16.67%) (0.080–0.400)	2/82 (2.44%) (0.150–0.300)	1/12 (8.33%) (0.056–0.320)	2/82 (2.44%) (0.205–0.490)	3/12 (25.00%) (0.135–0.350)	4/82 (4.88%) (0.050–0.240)	3/12 (25.00%) (0.208–0.502)	8/82 (9.76%) (0.160–0.400)	6/24 (25.00%) (0.050–0.360)	44/207 (21.25%) (0.156–0.475)
Qinghai	2/9 (22.22%) (0.014–0.068)	14/106 (13.21%) (0.092–0.215)	2/9 (22.22%) (0.103–0.196)	7/106(6.60%) (0.160–0.340)	2/9 (22.22%) (0.205–0.420)	3/106 (2.38%) (0.065–0.250)	2/9 (22.22%) (0.215–0.405)	24/106 (22.64%) (0.085–0.256)	0/7 (0.00%) (–)	0/54 (0.00%) (–)	0/7 (0.00%) (–)	0/54 (0.00%) (–)	1/7 (14.29%) (0.052–0.299)	3/54 (5.56%) (0.205–0.398)	1/7 (14.29%) (0.146–0.424)	3/54 (5.56%) (0.205–0.495)	3/16 (18.75%) (0.075–0.252)	27/160 (16.88%) (0.162–0.245)
Sichuan	3/8 (37.50%) (0.152–0.385)	7/85 (8.24%) (0.164–0.510)	3/8 (37.50%) (0.208–0.355)	6/85(7.06%) (0.056–0.250)	1/8 (12.25%) (0.052–0.150)	1/85 (1.18%) (0.210–0.285)	3/8 (37.50%) (0.056–0.145)	14/85 (16.47%) (0.152–0.465)	0/8 (0.00%) (–)	0/46 (0.00%) (–)	1/8 (12.25%) (0.220–0.352)	1/46 (2.17%) (0.155–0.264)	2/8 (25.00%) (0.034–0.092)	2/46 (4.35%) (0.095–0.160)	2/8 (25.00%) (0.065–0.158)	3/46 (6.52%) (0.168–0.314)	5/16 (31.25%) (0.252–0.416)	17/131 (12.98%) (0.075–0.176)
Total Pos/Tot (%)	8/29 (27.59%) (0.095–0.168)	44/316 (13.92%) (0.180–0.325)	7/29 (24.14%) (0.045–0.356)	22/316(6.96%) (0.155–0.320)	6/29 (20.69%) (0.105–0.250)	8/316 (2.53%) (0.040–0.346)	8/29 (27.59%) (0.250–0.503)	74/316 (23.42%) (0.039–0.308)	2/27 (7.14%) (0.165–0.290)	2/182 (1.10%) (0.045–0.116)	2/27 (7.41%) (0.205–0.486)	3/182 (1.65%) (0.085–0.405)	6/27 (22.22%) (0.081–0.196)	9/182 (4.95%) (0.125–0.362)	6/27 (2.22%) (0.054–0.218)	14/182(7.69%) (0.152–0.236)	14/56 (25.00%) (0.102–0.187)	88/498 (17.68%) (0.203–0.384)

CI, confidence interval

### Phylogenetic analysis and genotyping of PCV3 based on Cap genes

**Fig. 3:**
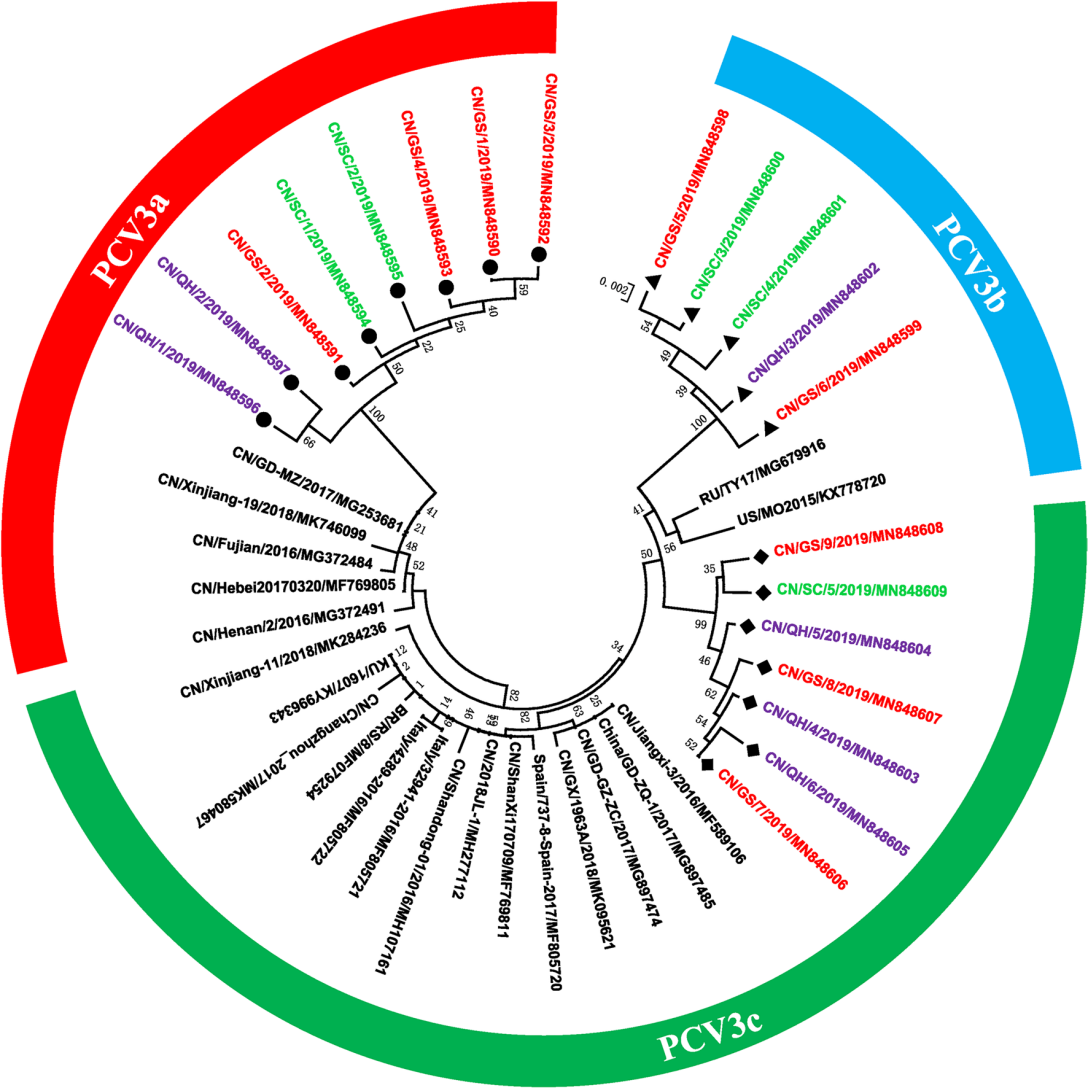
Phylogenetic analysis of porcine circovirus type 3 (PCV3) strains based on the Cap gene (ORF2). The phylogenetic tree was constructed from 20 distinct Cap sequences generated in this study and 21 sequences from known PCV3 strains, representing the different PCV3 subtypes. Different colours have been used in the circles to distinguish subtypes of PCV3 (red represents the PCV3a subtype, blue represents the PCV3b subtype, and green represents the PCV3c subtype). Bootstrap values expressed as percentages of 1000 replications are given at the branch points. The 20 newly identified Tibetan pig PCV3 strains PCV3a, PCV3b and PCV3c described in the present study are indicated by “filled circle”, “filled triangle” and “filled diamond”, respectively. The scale bar indicates nucleotide substitutions per site

The entire Cap genes of PCV3 strains in 88 positive samples were subjected to sequencing and used to confirm the subtypes of PCV3 in Tibetan pigs. Twenty different nucleotide sequences of Cap proteins were obtained from 88 PCV3-positive DNA samples. Sequence analysis identified 20 different viral strains, the Cap proteins of which shared 95.84–99.18% nucleotide identitiy (Table 3). They also shared 97.51–98.75% nucleotide identity with Chinese PCV3 reference strains, and 96.34–98.91% nucleotide identity with PCV3 reference strains from other countries. Phylogenetic analysis based on twenty nucleotide sequences of Cap proteins demonstrated that the 20 PCV3 strains formed three clades, including PCV3a (8/20, 40.00%), PCV3b (5/20, 25%) and PCV3c (7/20, 35.00%) (Figs. 2, 3 and Table 2). For PCV3 strains from the same geographic origin, the strains in the PCV3a subtype were divided into the same branches at three levels, while the strains in the PCV3b and PCV3c subtypes were divided into different branches at three levels. In all three provinces on the Qinghai-Tibet Plateau of China, the positivity rates of PCV3a and PCV3b at both the sample and farm levels in the samples from diarrheic Tibetan pigs were significantly higher than those in healthy samples. The positive rates of PCV3c in samples collected from pigs with diarrhoea at the sample level were significantly lower than those in healthy samples. From the 46 PCV3a-positive samples, eight different Cap genes were obtained, of which seven were from samples from pigs with diarrhoea, and one was from healthy a sample (CN/GS/4/2019/MN848593 (Table 2), located in one independent branch at three levels in the phylogenetic tree based on the Cap gene. Five different Cap genes were identified in 25 PCV3b-positive samples, of which four of them were from diarrheic animals and one from a healthy animal (CN/SC/4/2019/MN848601), which was also located in one independent branch. Seven different Cap genes were identified in 17 PCV3c-positive samples, of which three were from 8 diarrheic animals, and four were identified in 9 healthy samples (CN/QH/5/2019/MN848604, CN/GS/7/2019/MN848606, CN/GS/8/2019/MN848607, CN/GS/9/2019/MN848608), and these genes were divided into different branches at three levels.

**Table 3:** Sequence analysis of Cap genes of PCV3 strains

Selected strains (GenBank accession no.)	Cap gene		Complete genome
	Nucleotide	Amino acids	nucleotide
Identity of the PCV3 strains identified in our study	95.84–99.18%	87.86–99.52%	94.64–99.82%
Compared with Chinese PCV3 reference strains^a^	97.51–98.75%	91.52–97.10%	93.86–97.55%
Compared with PCV3 reference strains from other countries^b^	96.34–98.91%	91.52–97.10%	93.12–98.65%

^a^The GenBank accession no. of Chinese PCV3 reference strains: MG897474, MG253681, MG372484, MF769805, MK580467, MG372491, MK746099, MF589106, MK095621, MH277112, MH107161, MF769811, MK284236, and MG897485

^b^The GenBank accession no. of PCV3 reference strains from other countries: MG679916, MF805721, KY996343, MF079254, MF805722, MF805720, and KX778720

### Amino acid substitutions of Cap genes in PCV3 strains isolated from Tibetan pigs

**Fig. 4:**
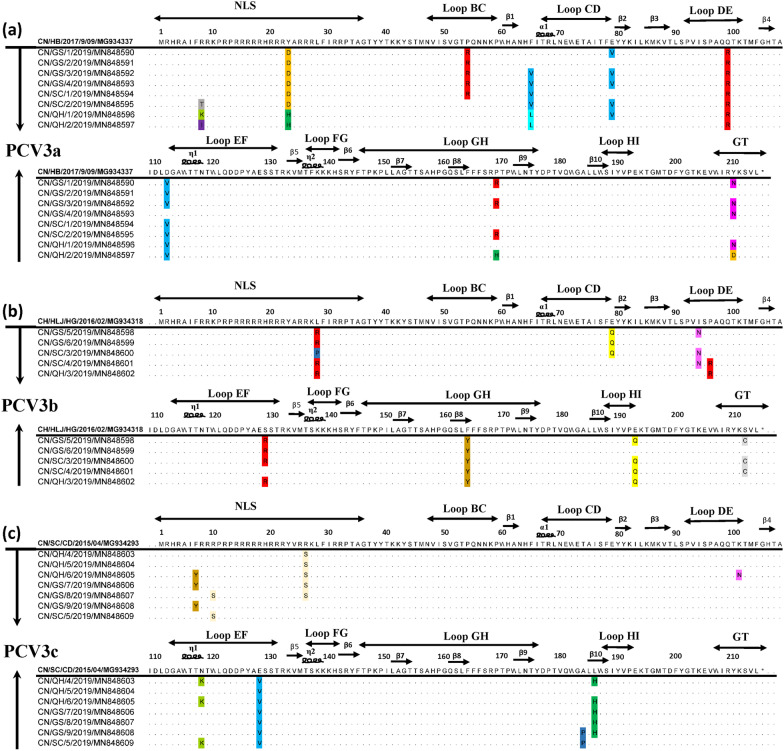
Alignment analysis of the amino acid sequences of the Cap protein between the identified porcine circovirus type 3 (PCV3) strains and the PCV3 reference strain (CH/HLJ/HG/2016/02/MG934318). **a–c** Represent the alignments of amino acid sequences of PCV3a, PCV3b and PCV3c

To identify amino acid substitutions in the Cap proteins of PCV3 strains isolated from Tibetan pigs, twenty deduced amino acid sequences of each genotype were selected and aligned with the reference sequence of the same genotype prevalent in China and obtained from GenBank. Amino acid sequence analysis of Cap proteins showed that the 20 different viral strains shared 87.86–99.52% identity (Table 3). As shown in Fig. 4, nine amino acid mutations were found in Cap proteins of PCV3a, and eight amino acid mutations were found in Cap proteins of the PVC3b and PVC3c subtypes. The amino acid substitutions in the Cap proteins occurred primarily in the 1–100 amino acid regions in PVC3a subtype (amino acid residues 8, 23, 54, 65, 79, 99). Mutations were found at sites 113, 169 and 210, while no mutation was observed at the site 113 in the strain obtained from healthy samples. In the PVC3b subtype, eight amino acid mutations were found sites 28, 79, 94, 96, 129, 164, 193 and 212, two mutation sites were in Loop DE region, while no mutation was observed at the site 129 in the strain obtained from healthy samples. There were three amino acid substitutions (7, 10 and 26) located in the functional domains of NLS among the PVC3c subtype strains in Tibetan pigs compared with the reference sequence. Two mutations (118 and 128) were found in the loop EF domain, and the other amino acid mutations were found at amino acid positions 101, 183 and 186. No mutation was observed at site 118 in the strains obtained from healthy samples.

### Phylogenetic analysis of the complete PCV3 genome

**Fig. 5:**
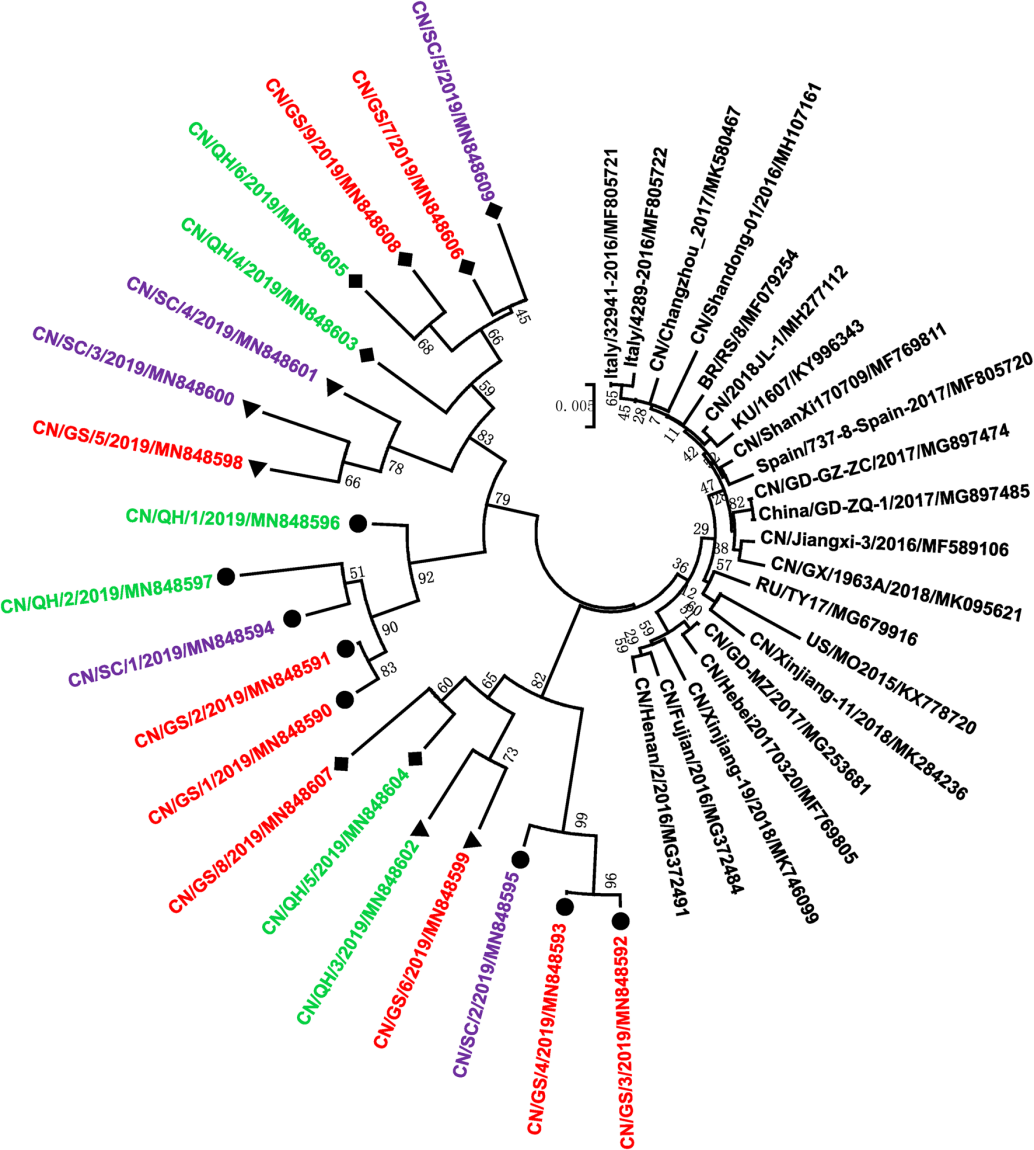
Phylogenetic analysis of porcine circovirus type 3 (PCV3) based on the whole-genome sequences. The phylogenetic tree was constructed by using the neighbour-joining method with MEGA 7.01 software (http://www.megasoftware.net). Bootstrap values were calculated with 1000 replicates. The number on each branch indicates bootstrap values. The 20 newly identified Tibetan pig PCV3 strains (GenBank accession no. MN848590-MN848609) PCV3a, PCV3b and PCV3c described in the present study are indicated by “filled circle”, “filled triangle” and “filled diamond”, respectively. Samples from Gansu, Sichuan and Qinghai are shown in red, violet, and green, respectively

To compare the complete genome sequences of twenty PCV3 strains found in Tibetan pigs, three amplified fragments from twenty PCV3 strains were sequenced and sequences information was submitted to the GenBank database under the accession numbers MN848590-MN848609. The PCV3 sequences isolated in our present study were used to construct a phylogenetic tree with sequences from 22 reference strains, obtained from GenBank, by the neighbour-joining method (Fig. 5). The whole genome sequence similarity ranged from 94.64 to 99.82% among the 20 different viral strains, and from 93.12 to 98.65% compared with reference sequences obtained from GenBank (Table 3). The complete genome sequence revealed that these strains formed one branch in the phylogenetic tree, and there were three subclusters found in the first-order branches (Fig. 5). However, there was crossover among different subtypes in some subclusters, PCV3a, PCV3b and PCV3a were in one subcluster, and PCV3b and PCV3a were in another subcluster. Only one subtype was found in the tertiary branches of the evolutionary tree (Fig. 5).

## Discussion

Several studies have previously shown that PCV3 has spread widely around the world, infecting mainly domestic pigs [11, 12, 16, 17, 25–27]. This virus has also been shown to have high prevalence in wild boars in Germany [12] and Sardinia [25]. In the present study, PCV3 was detected in Tibetan pigs for the first time. The prevalence of PCV3 in Tibetan pigs at the sample and farm levels was clearly lower than that in pigs from other provinces of China [8, 10, 15, 22, 26, 28], and other countries [27, 29, 30]. The Tibetan pig is a high-altitude pig breed and is highly economically important for the people living in those areas [31]. The cold and harsh environment where the Tibetan pig dwells might be a reason for the lack of reports about microbial infection in domestic animals [21]. However, there have been several reports of microbial infections reports in Tibetan pigs in recent years, such as pseudorabies virus [32], bufavirus [20, 33], porcine epidemic diarrhoea virus [34], hepatitis E virus [33] and porcine deltacoronavirus [21] infections. A common viral trait found among these viruses in Tibetan pigs is their lower prevalence than that in domestic pigs that live in low-altitude regions. This thus shows the importance of the influence of the species and environment of animals on microbial infection. Another study found that the prevalence of PCV3 in Tibetan pigs and domestic pigs was significantly higher than that a novel circovirus (PCV4) in Inner Mongolia [9] and Guangxi [23] provinces of China. Therefore, the prevalence of novel circovirus has gradually increased since it was first discovered.

Further systematic evaluation of the prevalence of PCV3 in Tibetan pigs showed that the prevalence varied among the three studied provinces on the Qinghai-Tibet Plateau of China. Furthermore, the prevalence of PCV3 at the farm level was higher than at the sample level, which is consistent with the discovery of PVC3 in other provinces of China [22, 23, 35], demonstrating the wide geographical distribution of PCV3 in China. The results of our present study suggested that PCV3 was prevalent in healthy samples; however, the prevalence was significantly lower than that in samples collected from pigs with diarrhoea, consistent with previous studies [18, 23, 24]. Co-infection of PCV3 with other virus have been reported in China, such as classical swine fever virus (CSFV), porcine reproductive and respiratory syndrome virus (PRRSV) [36] and porcine epidemic diarrhea virus (PEDV) [37], and the pathogenicity of PCV3 could be influenced by these co-infection pathogens in pigs. These findings suggested that other virus detection in Tibetan pigs should not be ignored, and PCV3 detection is essential in the asymptomatic pigs.

In previous studies, PCV3 was divided into three subtypes, namely, PCV3a, PCV3b and PCV3c based on phylogenetic analysis of the Cap gene [11, 12]. However, other research on PCV3 in China divided PCV3 into two subtypes, namely PCV3a and PCV3b, based on phylogenetic analysis of the whole genome of PCV3 [22], which demonstrated that the Cap gene analysis did not support the classification of PCV3 into different gene subtypes. The PCV3 strains in Tibetan pigs were also classified into the PCV3a, PCV3b and PCV3c subtypes based on the alignment of amino acid sequences of Cap proteins. Nevertheless, strains of the same subtype based on Cap protein were not grouped into the same subcluster in the phylogenetic tree based on the whole-genome sequence. These data indicated that mutations occurred in both Cap gene and other ORFs of PCV3, and mutation sites were much more in the other ORFs. So the sequence similarity of the whole genome was lower than the Cap gene among the different PCV3 strains. Additionally, it is important that the PCV3 arrangements are needed to validate this genotype classification. Moreover, a novel circovirus was discovered in the Inner Mongolia [9] and Guangxi [4] provinces of China in the last 2 years and named PCV4, this virus shared 36.9–73.8% sequence similarity with other circoviruses, showing that new mutations had occurred in these viruses. In addition to, the differences between the allocation of samples to each of the three genotypes using the Cap gene and full-length sequences reminds us the possibility of reassortment should be considered, which have been found in PCV2 [38]. Therefore, further investigation of genetic diversity should be carried out to determine whether this is recombination in PCV3.

In the present study, we found that the positive prevalence of PCV3a and PCV3b at both the sample and farm levels in the samples of diarrheic Tibetan pigs was significantly higher than that in healthy samples. The PCV3c positivity rates at the sample level in samples collected from pigs with diarrhoea were found to be significantly lower than those in healthy samples. The mutation rate of PCV3c was determined to be higher than that of PCV3a and PCV3b. Moreover, we found that there were more difference in Cap genes in PCV3 strains in samples collected from pigs with diarrhoeat than that in healthy samples. These results indicate that there are differences in pathogenicity among different subtypes of PCV3, PCV3a and PCV 3b are considered to be the cause of reproductive failure [11, 23, 39]. PCV3c was originally described as a putative cause of multisystemic inflammation in domestic pigs in previous studies [3]. Although the pathogenicity of PCV3a in domestic pigs and Tibetan pigs associated with genotype is consistent with the findings in this study, the genotype and host should not be neglected during surveys of the pathobiology of PCV3.

## Conclusion

In conclusion, the results of our present study show, for the first time, the wide prevalence of PCV3 infection in Tibetan pigs in China. The prevalence rates of different PCV3 subtypes in samples collected from pigs with diarrhoea indicated that genotype should not be neglected during survey of the pathogenicity of PCV3. Phylogenetic analyses suggested that the continuous evolution and adaptation of PCV3 to its hosts in a special environment should be further studied. These results broaden our understanding of the geographical lineage theory of PCV3. The results also provide additional information indicating that further systematic studies should be carried out to study the mechanisms of pathogenicity, transmission, evolution and effective vaccination against PCV3, and to develop vaccines for swine in high altitude regions.

## Data Availability

All data generated or analyzed during this study are included in this published article.
